# Research advancements in the Use of artificial intelligence for prenatal diagnosis of neural tube defects

**DOI:** 10.3389/fped.2025.1514447

**Published:** 2025-04-17

**Authors:** Maryam Yeganegi, Mahsa Danaei, Sepideh Azizi, Fatemeh Jayervand, Reza Bahrami, Seyed Alireza Dastgheib, Heewa Rashnavadi, Ali Masoudi, Amirmasoud Shiri, Kazem Aghili, Mahood Noorishadkam, Hossein Neamatzadeh

**Affiliations:** ^1^ Department of Obstetrics and Gynecology, School of Medicine, Iranshahr University of Medical Sciences, Iranshahr, Iran; ^2^ Department of Obstetrics and Gynecology, School of Medicine, Iran University of Medical Sciences, Tehran, Iran; ^3^ Shahid Akbarabadi Clinical Research Development Unit, Iran University of Medical Sciences, Tehran, Iran; ^4^ Neonatal Research Center, Shiraz University of Medical Sciences, Shiraz, Iran; ^5^ Department of Medical Genetics, School of Medicine, Shiraz University of Medical Sciences, Shiraz, Iran; ^6^ School of Medicine, Tehran University of Medical Sciences, Tehran, Iran; ^7^ School of Medicine, Shahid Sadoughi University of Medical Sciences, Yazd, Iran; ^8^ School of Medicine, Shiraz University of Medical Sciences, Shiraz, Iran; ^9^ Department of Radiology, School of Medicine, Shahid Rahnamoun Hospital, Shahid Sadoughi University of Medical Sciences, Yazd, Iran; ^10^ Mother and Newborn Health Research Center, Shahid Sadoughi University of Medical Sciences, Yazd, Iran

**Keywords:** artificial intelligence, prenatal diagnostics, machine learning, neural tube defects, ultrasound imaging

## Abstract

Artificial Intelligence is revolutionizing prenatal diagnostics by enhancing the accuracy and efficiency of procedures. This review explores AI and machine learning (ML) in the early detection, prediction, and assessment of neural tube defects (NTDs) through prenatal ultrasound imaging. Recent studies highlight the effectiveness of AI techniques, such as convolutional neural networks (CNNs) and support vector machines (SVMs), achieving detection accuracy rates of up to 95% across various datasets, including fetal ultrasound images, genetic data, and maternal health records. SVM models have demonstrated 71.50% accuracy on training datasets and 68.57% on testing datasets for NTD classification, while advanced deep learning (DL) methods report patient-level prediction accuracy of 94.5% and an area under the receiver operating characteristic curve (AUROC) of 99.3%. AI integration with genomic analysis has identified key biomarkers associated with NTDs, such as Growth Associated Protein 43 (GAP43) and Glial Fibrillary Acidic Protein (GFAP), with logistic regression models achieving 86.67% accuracy. Current AI-assisted ultrasound technologies have improved diagnostic accuracy, yielding sensitivity and specificity rates of 88.9% and 98.0%, respectively, compared to traditional methods with 81.5% sensitivity and 92.2% specificity. AI systems have also streamlined workflows, reducing median scan times from 19.7 min to 11.4 min, allowing sonographers to prioritize critical patient care. Advancements in DL algorithms, including Oct-U-Net and PAICS, have achieved recall and precision rates of 0.93 and 0.96, respectively, in identifying fetal abnormalities. Moreover, AI's evolving role in genetic research supports personalized NTD prevention strategies and enhances public awareness through AI-generated health messages. In conclusion, the integration of AI in prenatal diagnostics significantly improves the detection and assessment of NTDs, leading to greater accuracy and efficiency in ultrasound imaging. As AI continues to advance, it has the potential to further enhance personalized healthcare strategies and raise public awareness about NTDs, ultimately contributing to better maternal and fetal outcomes.

## Introduction

Artificial intelligence (AI) is transforming various aspects of medicine, including diagnostics, treatment planning, and patient care (1). AI algorithms improve diagnostic accuracy by analyzing extensive clinical data from electronic health records (EHRs) and medical imaging, facilitating the rapid and precise identification of diseases such as coronary artery disease and cancers (2, 3). AI in radiology improves image interpretation, enhances condition detection via computer-assisted diagnosis systems, and advances prenatal ultrasound diagnostics for fetal anomaly identification (4–6). Moreover, machine learning (ML) models are being developed for predictive analytics, enabling healthcare providers to evaluate patient outcomes based on historical data, which is especially beneficial for managing chronic diseases (7). AI's ability to integrate large datasets allows for the creation of personalized treatment plans tailored to individual genetic and clinical profiles, resulting in more effective therapies (8, 9). Additionally, AI supports clinicians with decision support systems that provide real-time recommendations based on current guidelines, which is crucial in urgent care settings for optimizing triage and resource allocation. The automation of routine administrative tasks through AI can mitigate physician burnout, allowing healthcare providers to focus more on patient care and ultimately enhancing job satisfaction. However, the integration of AI in healthcare raises ethical concerns related to data privacy, algorithmic bias, and the implications of machine decision-making (8, 10). This highlights the necessity for diverse training datasets to ensure equitable clinical outcomes. Despite these challenges, the potential of AI and ML to improve healthcare delivery is substantial, empowering healthcare professionals and contributing to a more efficient system (11, 12).

**Figure 1:**
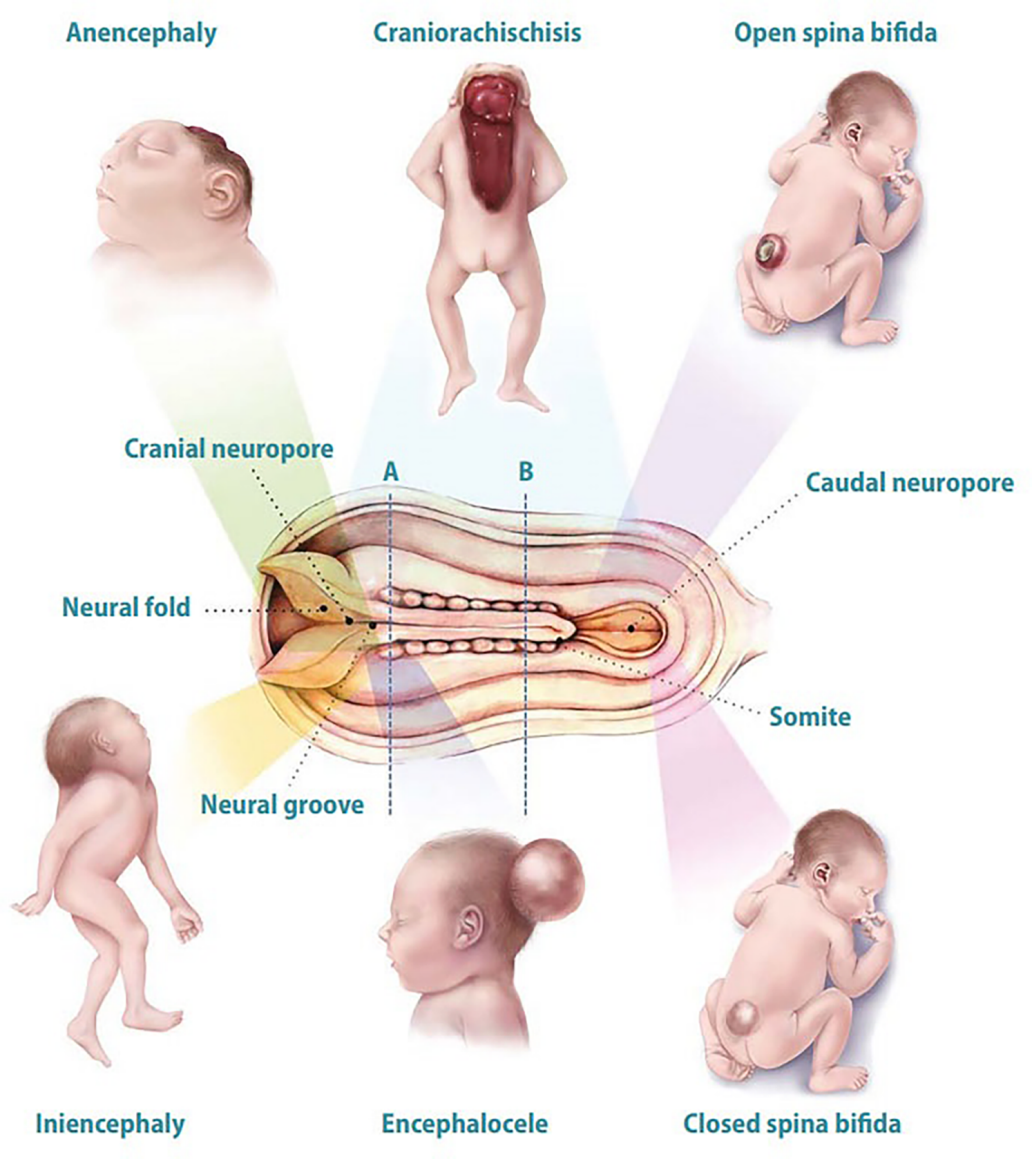
Overview of various NTDs in their developmental contexts. The NTDs depicted include anencephaly (absence of brain and skull), craniorachischisis (complete neural tube failure affecting both skull and spine), open spina bifida (exposed spinal column), closed spina bifida (defect with the spinal cord covered by skin but potential hidden neurological issues), encephalocele (protrusion of brain tissue through an abnormal skull opening), and iniencephaly (malformations of the skull and spine). Key developmental structures—neural groove, neural fold, cranial neuroport, caudal neuroport, and somite—are labeled to contextualize neural tube formation and associated defects. These conditions emerge during the critical phase of neural tube closure, highlighting the importance of proper embryonic development and potential disruptors. In the developing neural tube, area “A” refers to the dorsal aspect involved in patterning, while area “B” represents the ventral aspect where motor neuron progenitors are generated and brain and spinal cord vesicles begin to differentiate. This figure is adapted from Botto et al. (16).

Neural tube defects (NTDs) are major public health issues arising from incomplete closure of the neural tube during early embryonic development (13, 14). These multifactorial conditions, shaped by genetic and environmental factors, include serious birth defects like spina bifida and anencephaly (15, 16). Figure 1 illustrates the different types of NTDs within their developmental contexts. Their prevalence varies significantly by region due to genetic and environmental factors. Globally, the incidence of NTDs is approximately 2.1 per 100,000 (17), with prevalence rates between 0.3 and 199.4 per 10,000 births (18). Sub-Saharan Africa has the highest incidence at about 4.0 per 100,000 (17), while North America has the lowest at around 0.5 (19). Over the past two decades, NTD incidence has generally declined in about 89% of countries, thanks to improved public health initiatives like folic acid fortification. These trends highlight the urgent need for targeted public health strategies to promote folic acid supplementation among women of childbearing age, increasing awareness and access to this crucial preventive measure (17). The evolution of the field has ushered in the integration of AI, presenting a significant opportunity to enhance the detection, management, and comprehension of NTDs, which could transform maternal and child health interventions (20). AI has made notable strides in the early diagnosis of NTDs by employing ML algorithms that analyze complex datasets, including fetal ultrasound images and maternal health records, to uncover subtle patterns that indicate these conditions much earlier than traditional methods would allow (21, 22). ML techniques are being utilized in several vital areas, such as biomarker identification, predictive modeling, and the analysis of diverse datasets. A prominent application of machine learning lies in identifying biomarkers that facilitate early diagnosis, ultimately improving our understanding of the biological mechanisms underlying NTDs and enabling more targeted interventions (23). Furthermore, ML has been utilized to analyze large datasets, such as EHRs, to predict NTD occurrences based on maternal and environmental factors. A systematic review highlighted the potential of deep learning (DL) models to extract meaningful patterns from complex health data, significantly improving predictive accuracy in healthcare, particularly in prenatal care, where timely interventions can reduce risks associated with NTDs (24). Moreover, ML has been integrated into the study of organoids to simulate and analyze the effects of various genetic and environmental factors on neural tube development (25). This integration enhances the analysis of experimental data, leading to more robust conclusions about the causes and potential treatments for NTDs. Furthermore, the combination of ML with advanced imaging technologies has shown promise in enhancing the detection and classification of defects in embryonic development by identifying subtle morphological changes associated with NTDs, aligning with the broader trend of using ML to improve diagnostic accuracy and efficiency in medical imaging (24, 26).

This review examines advancements and future directions in AI applications for diagnosing NTDs. It assesses the effectiveness of various AI techniques for predicting NTDs and classifying related genetic mutations. The review explores methods for biomarker discovery in open NTDs, evaluating their potential to enhance prenatal diagnostics and clinical outcomes. It also highlights the role of AI-assisted ultrasound technology in improving detection accuracy and integrating AI with systems biology to enhance our understanding of NTDs. Innovations in precision medicine, including personalized dietary recommendations for prospective parents and AI-driven health communication strategies for raising awareness about NTD prevention, are emphasized. The review concludes with future research directions, underscoring the need for collaboration between technology developers and clinicians while addressing the ethical implications of AI in this critical area of healthcare.

## An overview of machine learning and deep learning

AI, ML, and DL constitute a hierarchical framework of computational intelligence, each with unique methodologies and applications (1). AI is the broad framework for creating intelligent systems that mimic human cognitive functions like problem-solving and learning. ML, a subset of AI, allows systems to learn from data and experiences without explicit programming, using techniques such as supervised, unsupervised, and reinforcement learning (27). DL, a subset of ML, employs multi-layered neural networks to automatically extract features from large datasets. This complexity requires substantial computational power, often provided by Graphics Processing Units (GPUs), which outperform traditional Central Processing Units (CPUs) in parallel processing. While ML is suitable for simpler tasks and smaller datasets, DL excels in complex applications like image and speech recognition due to its advanced architecture. The rise of GPUs and other hardware accelerators has significantly enhanced the speed of training and efficiency of inference in DL (28–30). ML encompasses various algorithms that derive insights and make predictions across fields such as agriculture, healthcare, finance, and e-commerce, with performance heavily reliant on the quality and quantity of training data. In contrast, DL utilizes neural networks, including convolutional neural networks (CNNs) for image processing and recurrent neural networks (RNNs) for sequential data analysis, to handle complex data types (1, 31). Recent advancements in DL, especially in transformers and self-attention mechanisms, have significantly improved capabilities in tasks like language translation, making DL crucial for high-accuracy applications such as autonomous driving and robotics. The integration of ML and DL techniques enhances performance on complex problems, as seen in hybrid models that combine CNNs and long short-term memory networks (LSTMs) for text classification (32, 33). Despite their different methodologies, the interplay between ML and DL creates a robust framework for tackling complex challenges, with ongoing research necessary to address computational demands and model interpretability, optimizing these technologies for broader applications (34). AUROC is a vital metric for evaluating AI algorithms, particularly in binary classification tasks, as it measures a model's ability to distinguish between positive and negative classes across various thresholds, providing insights into sensitivity and specificity. An AUROC of 1 indicates perfect classification, while 0.5 suggests no discriminative ability, akin to random guessing. This metric is especially useful in scenarios with imbalanced class distributions, allowing for a more nuanced comparison of algorithms beyond simple accuracy. Incorporating AUROC into the evaluation process helps researchers and practitioners better understand the trade-offs associated with their AI models, ultimately enhancing decision-making capabilities (35).

## Use of AI in prenatal diagnostics

AI, particularly through ML and DL, is revolutionizing prenatal diagnostics by significantly enhancing the accuracy and efficiency of fetal assessments. These technologies are increasingly applied in various facets of prenatal care, such as screening for congenital anomalies, predicting pregnancy complications, and improving ultrasound imaging (21, 36, 37). AI algorithms, especially DL models, excel at analyzing ultrasound images to identify fetal anomalies that may be overlooked by human operators. For example, CNNs have been utilized to standardize fetal anatomy assessments, which minimizes inter-operator variability and boosts diagnostic consistency (38, 39). This advancement allows for more precise evaluations of conditions like congenital heart defects and kidney anomalies. Furthermore, AI has markedly improved non-invasive prenatal testing (NIPT) methods by elevating detection rates of chromosomal abnormalities through the analysis of complex datasets from maternal blood samples, offering safer diagnostic options for expectant mothers (40, 41). Moreover, ML models are employed to develop predictive analytics that can identify pregnancies at risk for complications such as preterm birth and preeclampsia by examining various factors, including maternal history and biophysical parameters, thus enabling personalized care strategies. AI technologies also support continuous monitoring of maternal and fetal health parameters through wearable devices that track vital signs, providing immediate alerts for any deviations and facilitating timely medical interventions (42, 43).

Thatoi et al. recently conducted a systematic review and meta-analysis on the effectiveness of AI algorithms for detecting congenital fetal abnormalities, particularly in the heart and brain. Analyzing data from 243,456 pregnancies, they found that DL and ML models exhibited high diagnostic performance, with sensitivity between 82% and 99% and specificity from 78% to 99%. Their analysis, utilizing the DerSimonian-Laird technique and Summary Receiver Operating Characteristic (SROC) plots, achieved a significant area under the SROC curve of 0.960, indicating a strong ability of these AI algorithms to distinguish between abnormal and normal cases across the included studies. Individual studies reported AUROC curves ranging from 0.85 to 0.95, demonstrating good to excellent discrimination. These findings highlight the potential of AI algorithms, especially CNNs, to deliver reliable diagnostic predictions, potentially reducing infant mortality and improving treatment outcomes for affected pregnancies (44). Moreover, Spahić et al. (45) advanced the integration of AI in diagnosing fetal neurological impairment disorders with their TRUEAID system. This innovative approach combines the finely tuned Kurjak Antenatal Neurodevelopmental Test (KANET), which utilizes four-dimensional ultrasound technology to assess fetal neurological development during the critical third trimester, with a convolutional neural network to create an AI-powered ultrasound module for automated diagnostic support. The study reports an accuracy of 93.83%, highlighting the system's potential for early detection of conditions like cerebral palsy, epilepsy, and autism spectrum disorder (45). The KANET, developed over the past 15 years, evaluates fetal behavior and movement patterns through a brief 15–20 min ultrasound session, meticulously recording specific criteria such as head, hand, and foot movements, eye movements, facial expressions, and the continuity of complex movements. The research emphasizes the importance of early diagnosis for timely intervention and illustrates the viability of AI technologies in enhancing fetal neurobehavioral assessments. This pilot study lays the groundwork for AI-based diagnostics, with significant implications for improving outcomes for affected children and expanding access to advanced diagnostic capabilities in various healthcare settings (46, 47).

## Use of AI for early detection of neural tube defects

AI offers a transformative approach to prenatal diagnostics, but challenges persist, including the requirement for large labeled datasets and the integration of AI tools into clinical practice. Still, AI holds significant potential for enhancing early detection and intervention for NTDs. Table 1 provides an overview of AI applications for the early detection and prediction of NTDs. Mahalle et al. (22) investigated the application of ML techniques, specifically CNNs and gradient boosting machines (GBMs), for the early detection of birth defects. The study emphasized the importance of early identification of these defects to facilitate timely intervention. By utilizing a diverse range of datasets, including fetal ultrasound images, genetic information, maternal health records, and demographic details, the authors trained their ML models on labeled data containing accurate diagnoses of various birth defects. The proposed methodology not only achieved high accuracy in detecting NTDs, fetal heart abnormalities, and chromosomal issues but also aimed to enhance understanding of the biological processes underlying these conditions. The user-friendly interface designed for healthcare professionals further supported its practical implementation, enabling them to provide prompt guidance and assistance to expecting parents (22). Vahedifard et al. (51) conducted a narrative review on the application of AI and ML techniques in fetal brain magnetic resonance imaging (MRI). The study highlights the prevalence of central nervous system abnormalities in fetuses, which occur in approximately 0.1% to 0.2% of live births and 3% to 6% of stillbirths, emphasizing the importance of early detection and categorization of these conditions. The authors discuss various AI models, primarily CNNs and U-Net architectures, that have been employed for automating the processing of anatomical fetal brain MRI. Notably, some models demonstrated accuracy rates exceeding 95%. The review also outlines the capabilities of AI in tasks such as gestational age prediction, fetal brain extraction and segmentation, and placenta detection. Furthermore, the study addresses the use of classification algorithms, including K-nearest neighbor and random forest, for identifying brain pathologies. The authors stress the need for large-scale, labeled datasets to enhance the efficacy of DL methods, as well as the importance of shared fetal brain MRI datasets due to the limited availability of such images. They conclude by highlighting the necessity for healthcare professionals, particularly neuroradiologists, general radiologists, and perinatologists, to understand the role of AI in improving fetal brain MRI diagnostics (51).

**Table 1:** Overview of AI applications in early detection and prediction of NTDs.

Study	Focus	Techniques used	Key findings and accuracy	Implications
Wang et al. (48)	Prediction of NTDs	SVM, RFE	SVM models predicted NTDs with 71.50% training accuracy	Suggests need for robust datasets and optimization of predictive models
Aguiar-Pulido et al. (49)	Genetic variations in spina bifida	Machine learning	Discovered function-altering variants associated with spina bifida	Informs targeted preventative strategies and personalized medicine
Mustafa et al. (50)	Gene polymorphism and folic acid	Grey wolf optimizer-assisted deep learning	Achieved 99.5% accuracy in predicting risk factors for NTDs	Highlights the importance of integrating genetic and nutritional factors
Karthik et al. (23)	Genetic markers for NTDs	RFE and differential gene expression	Identified critical biomarkers related to myelomeningocele	Enhances prenatal diagnostics for at-risk pregnancies
Weaver et al. (20)	Neurogenic bladder dysfunction	Ensemble models	Achieved 70% accuracy; highlights need for further enhancements	Indicates potential for improving NTD-related assessments
Vahedifard et al. (51)	Fetal brain MRI analysis	CNNs, U-Net architectures	Achieved accuracy rates over 95% in automating fetal brain MRI processing	Highlights the need for large, labeled datasets for improved diagnostics
Mahalle et al. (22)	Early detection of NTDs	CNNs, GBMs	High accuracy in detecting NTDs and other birth defects using diverse datasets	Supports timely intervention and enhances understanding of biological processes
Qi et al. (52)	Deep learning for NTDs	Deep learning models	Patient-level prediction accuracy of 94.5% and AUROC of 99.3%	Supports the use of heatmaps for assessing anomaly severity

Abbreviations: AI, artificial Intelligence; NTDs, neural tube defects; CNNs, convolutional neural networks; GBMs, gradient boosting machines; SVM, support vector machine; RFE, recursive feature elimination; AUROC, area under the receiver operating characteristic curve.

## Use of AI in prediction and assessment of severity of neural tube defects

Techniques like Support Vector Machines (SVM) and Recursive Feature Elimination (RFE) are effectively employed to analyze genetic and demographic data, enhancing the understanding and prediction of NTDs such as spina bifida (23). SVM classifies NTD occurrences by merging genetic and demographic datasets, clarifying risk factors for these birth defects (49). RFE refines feature selection to identify the most significant genetic markers. This integration improves model accuracy and informs public health initiatives and preventive measures for at-risk populations. The research spans public health, genetics, and maternal-fetal health, highlighting the importance of interdisciplinary approaches to complex health issues (53, 54). Current models show some predictive ability, but further research is necessary to refine methods and incorporate additional risk factors for better prenatal care outcomes. Wang et al. demonstrated that SVM models predicted NTDs with 71.50% accuracy on training datasets and 68.57% on testing datasets, emphasizing the need for robust datasets to enhance predictive performance. While these accuracy rates indicated potential for classifying and forecasting NTD rates at the village level, significant room for improvement remained. Future research should have focused on optimizing model parameters, exploring additional features, and integrating other ML algorithms (48). In contrast, Weaver et al. (20) advanced DL techniques for assessing neurogenic bladder dysfunction in spina bifida patients. Their ensemble model combined volume-pressure recordings—measuring urine volume and bladder pressure—with fluoroscopic imaging data, achieving 70% accuracy (95% CI 66%, 76%). However, a weighted kappa of 0.54 signifies only moderate agreement between the model's predictions and clinical evaluations, indicating substantial improvement is needed. The scale ranges from −1 (complete disagreement) to 1 (perfect agreement). These results underscore the importance of refining and validating the DL model to enhance its clinical applicability and reliability. With the clinical model showing only 61% accuracy and a weighted kappa of 0.37, the findings highlight the advantages of DL over traditional methods for more effectively assessing bladder dysfunction severity in this vulnerable population (20). Moreover, a 2025 study by Qi et al. highlighted DL's immense potential, reporting a patient-level prediction accuracy of 94.5% and an AUROC of 99.3% for various NTDs, with models generating heatmaps to assist clinicians in evaluating anomaly severity (52). Further exploration by Karthik et al. in 2022 employed RFE to identify critical genetic markers linked to open NTDs, emphasizing the multifactorial nature of NTDs and the necessity of integrating genetic insights into predictive models to enhance their accuracy and applicability in clinical settings (23). This holistic approach underscores the importance of multifaceted strategies in advancing the predictive power of models for complex health conditions such as NTDs.

## Integration of AI with biomarkers for detection of neural tube defects

AI models excel at analyzing extensive genomic datasets to identify various genetic variants, such as single-nucleotide variations (SNVs) and copy number variants (CNVs). These models enhance the sensitivity and specificity of variant calling, which is crucial for pinpointing mutations linked to congenital disorders (55). Tools like SpliceAI and MMSplice are often integrated into AI frameworks to prioritize harmful variants that may contribute to NTDs (56). Researchers at Weill Cornell Medicine have applied a ML approach to explore genetic variations associated with spina bifida, a type of NTD. By examining the genomes of 149 individuals with spina bifida and 149 healthy controls, they discovered function-altering variants in genes that differentiate affected individuals from those without the condition. The study highlighted significant pathways related to glucose and lipid metabolism, which are vital for cellular energy and influenced by maternal health factors like diabetes and obesity, known risk factors for NTDs. The findings demonstrate the efficacy of ML algorithms in classifying genetic data, enabling the identification of genes that distinguish spina bifida cases from controls, thus minimizing biases present in traditional studies. These insights could inform targeted preventative strategies, including personalized nutritional advice for couples planning to conceive. Moreover, the researchers aim to establish an international consortium to expand their investigation, enhancing the understanding of the genetic basis of spina bifida across diverse populations and promoting personalized medicine approaches to prevent this serious condition (49). Furthermore, a study by Karthik et al. (23) employed a ML technique, specifically RFE and differential gene expression analysis, to identify key biomarkers related to open NTDs, particularly myelomeningocele, by analyzing amniotic fluid datasets (GSE4182 and GSE101141). The research identified four significant biomarkers: Growth Associated Protein 43 (GAP43), Glial Fibrillary Acidic Protein (GFAP), Repetin (RPTN), and CD44, which are involved in critical processes such as axon growth, astrocyte differentiation, and neuroinflammation. The findings were validated using various binary classifiers, with logistic regression achieving an accuracy of 86.67% and an AUC-ROC of 0.90 in distinguishing affected from healthy samples. GAP43 and GFAP are particularly important for early diagnosis due to their roles in neurodevelopment and response to central nervous system injuries, with elevated levels indicating abnormal neurodevelopment and astroglial activation, respectively. This research highlights the potential of these biomarkers to improve prenatal diagnostic methods for conditions like myelomeningocele, enabling healthcare providers to identify at-risk pregnancies earlier and facilitating timely interventions for better outcomes in newborns (23). In their 2021 study, Mustafa et al. investigated the relationship between gene polymorphism and folic acid interactions in the context of NTDs. The research highlighted the significance of the minimum folate carrier (MFC A80G) gene polymorphism, particularly the homozygous mutant type (GG) genotype, alongside maternal folic acid consumption in reducing the prevalence of NTDs. Utilizing a grey wolf optimizer-assisted deep recurrent neural network, the authors achieved an impressive accuracy rate of 99.5% in predicting the associations between these factors. Their findings indicated a remarkably low error ratio of 0.015%, underscoring the effectiveness of combining genetic information with nutritional factors to mitigate the risk of NTDs in offspring (50). Folic acid plays a crucial role in reducing the risk of NTDs, including spina bifida and anencephaly, during pregnancy. As a vital B vitamin, it is essential for DNA synthesis, repair, and methylation—processes critical for early fetal development. Once converted to tetrahydrofolate (THF) in the body, folic acid supports nucleotide synthesis, aiding in the formation and closure of the neural tube (57). Research indicates that women who consume sufficient folic acid before and during early pregnancy can significantly lower their risk of NTDs (13, 14).

The integration of ML with systems biology is transforming the study of complex genetic conditions like spina bifida by allowing researchers to analyze genetic data in conjunction with environmental factors (49). This multidisciplinary approach enhances the understanding of conditions NTDs, which are influenced by both genetic predispositions and environmental exposures. By utilizing ML algorithms to process large datasets, researchers can effectively identify at-risk populations and develop targeted interventions, overcoming challenges faced by genome-wide association studies (GWAS) in detecting subtle genetic signals associated with rare conditions (58, 59). Ultimately, the goal is to create personalized prevention strategies tailored to individual families, allowing healthcare providers to offer customized advice and interventions that could reduce the risk of spina bifida and other NTDs. As ML continues to evolve, future research will likely focus on sophisticated models that incorporate various datasets, further improving predictive capabilities and deepening our understanding of complex genetic conditions (49). Overall, these studies underscores the transformative role of ML in biomedical research, particularly in identifying critical biomarkers for complex conditions such as open NTDs and advancing personalized maternal-fetal healthcare.

## Integration of AI in prenatal ultrasound imaging

Currently, there are relatively few studies utilizing AI to predict NTDs, with most existing models focusing on binary classification (normal vs. abnormal) or identifying general intracranial image patterns (60, 61). However, AI-assisted ultrasound technology is significantly enhancing the detection of NTDs and other fetal anomalies through various mechanisms. One of the most notable advancements is in diagnostic accuracy, where AI algorithms have achieved sensitivity and specificity rates as high as 88.9% and 98.0%, respectively. These rates surpass those of traditional methods, which report lower sensitivity at 81.5% and specificity at 92.2% (24, 26). Moreover, AI systems automate routine tasks, such as capturing standard image planes and measuring biometric parameters, which has reduced median scan times from 19.7 min to 11.4 min, thereby easing the cognitive burden on sonographers and allowing them to concentrate on other critical patient care aspects (26, 62). This streamlining leads to enhanced workflow efficiency, enabling sonographers to conduct smoother examinations with fewer interruptions and fostering better patient interactions. Moreover, AI provides real-time quality assurance during ultrasound examinations, offering immediate feedback to ensure that images meet diagnostic standards and mitigating human judgment errors. It also addresses traditional ultrasound limitations, such as fetal movement and inter-observer variability, by improving measurement consistency and anomaly detection under less-than-ideal conditions. Beyond NTDs, AI applications extend to assessing other fetal conditions, like congenital heart disease and brain abnormalities, with high accuracy in distinguishing normal from abnormal fetal brain structures (39, 63). This integration of AI into ultrasound technology represents a transformative advancement in prenatal care, significantly improving the screening and diagnosis of fetal anomalies and ultimately leading to better outcomes for affected infants and families. As research progresses, further advancements are anticipated, paving the way for fully automated systems and enhanced collaboration between AI developers and medical professionals. Overall, AI is transforming obstetric ultrasound by improving diagnostic capabilities, optimizing workflows, and facilitating timely medical interventions, ultimately enhancing prenatal care and outcomes for affected infants and families (4, 24).

Table 2 showcases advancements in DL algorithms for diagnosing fetal abnormalities, such as NTDs, using ultrasound and MRI, highlighting their potential to enhance diagnostic accuracy and efficiency in clinical settings. Recent studies emphasize notable progress in DL techniques for classifying and detecting fetal brain abnormalities through these imaging modalities. Chen et al. (62) developed an enhanced convolutional neural network algorithm called Oct-U-Net, aimed at improving the automatic recognition and diagnosis of fetal spina bifida in three-dimensional (3D) ultrasound images. This research involved 3,300 pregnant women who underwent 3D ultrasound examinations, with Oct-U-Net evaluated using various metrics such as recall rate, precision rate, pixel accuracy (PA), mean intersection over union (MIoU), and mean standard error. The findings revealed that Oct-U-Net outperformed both the fully convolutional network (FCN) and the original U-Net algorithm, achieving recall and precision rates of 0.93 and 0.96, respectively, alongside a PA of 0.949 and an MIoU of 0.917. Moreover, Oct-U-Net demonstrated a notably lower mean standard error of 4.1243 and a reduced average running time of 12.15 s compared to other algorithms, suggesting its superior diagnostic capabilities for fetal spina bifida in 3D ultrasound imaging (67). Xie et al. (64) examined the feasibility of DL algorithms on a large dataset from a hospital database, which included 10,251 normal and 2,529 abnormal pregnancies, ultimately analyzing 15,372 normal and 14,047 abnormal ultrasound images. Their algorithms achieved impressive segmentation precision, recall, and Dice's coefficient of 97.9%, 90.9%, and 94.1%, respectively, with an overall classification accuracy of 96.3%, sensitivity of 96.9%, and specificity of 95.9% (64). Lin et al. (65) introduced the Prenatal ultrasound diagnosis Artificial Intelligence Conduct System (PAICS), utilizing a dataset of 43,890 images and 169 videos from 16,297 pregnancies. PAICS demonstrated robust diagnostic performance in identifying ten types of intracranial abnormalities, achieving macro- and microaverage areas under the receiver-operating-characteristics curve (AUC) of 0.933 and 0.977 for internal validation, respectively (65). Chowdhury et al. (66) developed StackFBAs, a novel DL framework for detecting fetal brain abnormalities from MRI images, achieving an overall accuracy of 80% and incorporating federated learning techniques (66). Qi et al. (52) advanced the field further with a model focused on detecting central nervous system anomalies, achieving a patient-level prediction rate of 94.5% and an AUROC of 99.3% (52). These studies collectively illustrate the transformative potential of DL in prenatal diagnostics, significantly enhancing healthcare delivery and patient outcomes.

**Table 2:** Summary of studies on advances in deep learning for fetal imaging.

Study	Methodology	Dataset Details	Key Findings
Xie et al. (64)	Deep learning algorithms for classification of fetal brain ultrasound images	10,251 normal and 2,529 abnormal pregnancies; 15,372 normal and 14,047 abnormal images analyzed	Segmentation precision: 97.9%, recall: 90.9%, DICE: 94.1%; overall accuracy: 96.3%, sensitivity: 96.9%, specificity: 95.9%
Chen et al. (62)	Enhanced convolutional neural network (Oct-U-Net)	3,300 pregnant women, 3D ultrasound images	Outperformed FCN and original U-Net—Recall rate: 0.93—Precision rate: 0.96—Pixel accuracy (PA): 0.949—Mean intersection over union (MIoU): 0.917—Mean standard error: 4.1243—Average running time: 12.15 s
Lin et al. (65)	Development of the Prenatal ultrasound diagnosis Artificial Intelligence Conduct System (PAICS)	43,890 images from 16,297 pregnancies; 169 videos from 166 pregnancies	AUC of 0.933 (macro) and 0.977 (micro) for internal validation; performance comparable to expert sonologists
Chowdhury et al. (66)	StackFBAs deep learning framework for detecting fetal brain abnormalities from MRI images	Utilized a Greedy-based Neural Architecture Search to generate 94 CNN architectures	Overall accuracy: 80%, F1-score: 78%, sensitivity: 76%, specificity: 78%; incorporated federated learning techniques
Qi et al. (52)	Deep learning model for detection and classification of fetal CNS anomalies using ultrasound imaging	Multi-center dataset focusing on four anomalies: anencephaly, encephalocele, holoprosencephaly, rachischisis	Patient-level prediction rate: 94.5%, AUROC: 99.3%; improved diagnostic accuracy with heatmaps for visual cues

Abbreviations: AUC, area under the curve; CNN, convolutional neural network; DICE, sørensen-dice coefficient; FCN, fully convolutional network; MIoU, mean intersection over union; PA, pixel accuracy; AUROC, area under the receiver operating characteristic; F1-score, harmonic mean of precision and recall.

## Future directions

The integration of AI into genetic research is transforming NTD prevention by enabling personalized strategies for prospective parents. AI allows for the thorough analysis of genetic data to identify mutations and variations that increase the risk of NTDs, while also factoring in environmental influences. This comprehensive approach enables healthcare providers to offer tailored recommendations, such as personalized nutritional advice on folic acid, which effectively reduces NTD risks. The goal is to empower families by considering their unique genetic backgrounds, lifestyle choices, and dietary needs. Moreover, AI enhances traditional GWAS to overcome challenges associated with the rarity of NTDs, leading to more robust findings (68–70). As AI technology advances, its role in genetic research is expected to expand, promoting interdisciplinary collaboration that translates findings into clinical practice and paving the way for personalized NTD prevention strategies.

AI-generated health messages are proving effective in raising public awareness about NTD prevention, particularly regarding the importance of folic acid. Research shows that these messages often match or exceed the clarity and effectiveness of human-created content, as seen in studies like the Folic Acid Message Engine (71, 72). This technology produces tailored messages that effectively engage specific demographics, ensuring broader reach on social media. Moreover, AI can generate a consistent flow of fresh content, addressing challenges in maintaining long-term health communication campaigns. By providing timely reminders and educational materials for prospective parents, AI helps close significant knowledge gaps about folic acid's role in reducing NTD risks. As AI technology evolves, its application in health communication may grow, potentially leading to more sophisticated algorithms that analyze audience responses in real-time, while also emphasizing the importance of ethical considerations. Overall, AI-generated health messages offer a valuable opportunity to enhance public understanding of NTD prevention and improve health outcomes for at-risk communities.

## Conclusions

The integration of AI into the study and prevention of NTDs represents a significant advancement in research and clinical practice. Studies showcase various applications of these technologies, such as predictive modeling, genetic mutation identification, biomarker discovery, and AI-assisted imaging, which enhance diagnostic accuracy, patient care, and personalized prevention strategies based on individual genetic and environmental factors. As research evolves, the potential for precision medicine grows, allowing healthcare providers to offer targeted recommendations based on genetic predispositions and lifestyle choices. Furthermore, AI-driven health awareness campaigns can improve public understanding of NTD prevention, guiding informed decisions by prospective parents. Moving forward, combining ML techniques with traditional medical practices will enhance our understanding of complex conditions like spina bifida and promote innovative solutions. Collaborative efforts across disciplines will be essential for translating these advancements into practical benefits for at-risk patients. This ongoing exploration underscores AI's crucial role in the future of maternal-fetal medicine and public health initiatives aimed at reducing NTD incidence.
